# The Infiltration of Silver Nanoparticles into Porous Silicon for Improving the Performance of Photonic Devices

**DOI:** 10.3390/nano12020271

**Published:** 2022-01-15

**Authors:** Rehab Ramadan, Raúl J. Martín-Palma

**Affiliations:** 1 Departamento de Física Aplicada, Universidad Autónoma de Madrid, 28049 Madrid, Spain; rauljose.martin@uam.es; 2 Department of Physics, Faculty of Science, Minia University, Minia 61519, Egypt; 3 Instituto Universitario de Ciencia de Materiales Nicolás Cabrera, Universidad Autónoma de Madrid, 28049 Madrid, Spain

**Keywords:** porous silicon, silver nanoparticles, reflectance, impedance spectroscopy, photoresponse

## Abstract

Hybrid nanostructures have a great potential to improve the overall properties of photonic devices. In the present study, silver nanoparticles (AgNPs) were infiltrated into nanostructured porous silicon (PSi) layers, aiming at enhancing the optoelectronic performance of Si-based devices. More specifically, Schottky diodes with three different configurations were fabricated, using Al/Si/Au as the basic structure. This structure was modified by adding PSi and PSi + AgNPs layers. Their characteristic electrical parameters were accurately determined by fitting the current–voltage curves to the non-ideal diode equation. Furthermore, electrochemical impedance spectroscopy was used to determine the electrical parameters of the diodes in a wide frequency range by fitting the Nyquist plots to the appropriate equivalent circuit model. The experimental results show a remarkable enhancement in electrical conduction after the incorporation of metallic nanoparticles. Moreover, the spectral photoresponse was examined for various devices. An approximately 10-fold increment in photoresponse was observed after the addition of Ag nanoparticles to the porous structures.

## 1. Introduction

Crystalline silicon is likely to remain the dominant semiconductor for the development of photovoltaic solar cells. However, the indirect bandgap and optical losses of silicon limit its performance as a photoactive material [1,2]. There are several methods to enhance the optoelectronic properties of crystalline silicon, such as fabricating multi-bandgap devices [3,4], adjusting the bandgap of silicon [5], or growing anti-reflective coatings [6,7] such as nanostructured porous silicon (PSi), which is commonly fabricated by the electrochemical etch of Si wafers [5,8].

An increase in porosity of the PSi layers leads to a reduction in the effective index of refraction. At the same time, increased porosity results in reduced crystallite size and increased transparency. PSi can be described as a mixture of silicon nanocrystallites, amorphous silicon, and air [9,10]. Moreover, its optical behavior depends on the porosity and thickness of the porous layer [11,12,13]. As a consequence of the formation of the nanopores in the heavily doped Si regions, the increase in the thickness of the PSi layer leads to electrical losses that negatively affect the overall photovoltaic characteristic parameters. 

Incorporating metallic nanoparticles can be used for optical enhancement. However, the density, shape, and size of metallic nanoparticles have to be optimized, to overcome the increase in reflectance. From another perspective, using hybrid nanostructures is another strategy to enhance the optoelectronic performance of photovoltaic devices. The growth of metallic nanoparticles into porous structures maintains the optical enhancement and limits the electrical reduction in the active photovoltaic layer.

The accurate determination of the electrical properties of photovoltaic devices is of foremost importance to predict and optimize their overall performance. For instance, photocarrier generation and recombination mainly depend on the doping concentration of the active material and the carrier diffusion length. Moreover, the electrical conduction of the active layer and the setup of the bottom and top contacts are critical for lowering the series resistance and increasing the shunt resistance (parasitic resistances have a significant effect on the fill factor of solar cells). Direct current (DC) and alternating current (AC) electrical measurements are suitable to study the conduction mechanism and electrical parameters of electronic devices. As such, DC analysis provides some key electrical parameters from current–voltage (I–V) curves, including reverse saturation current, parasitic resistances, ideality factor, and barrier height. Furthermore, AC analysis by electrochemical impedance spectroscopy (EIS) provides the electrical conduction properties of the interfaces of a device in a wide frequency range by establishing an equivalent circuit model [14].

In the current study, the impact on optical properties of the infiltration of silver nanoparticles (AgNPs) in PSi layers was studied. Additionally, a comparison was drawn between the electrical properties of three different metal–semiconductor junctions and analyzed in detail. The three junctions under analysis were Al/Si/Au, Al/Si + PSi/Au, and Al/Si + PSi + AgNPs/Au. DC analysis was executed by measuring the I–V curves and fitting the results to the non-ideal diode equation. Furthermore, EIS was used to establish the AC analysis in a wide range of frequencies from 1 Hz to 1 MHz, in addition to fitting the data to the most suitable equivalent circuit model, to summarize the electrical parameters between the interfaces. Additionally, the spectral photoresponse of Al/Si + PSi/Au and Al/Si + PSi + AgNPs/Au Schottky junctions was acquired at zero bias, in the wavelength range from 300 nm to 1200 nm.

## 2. Materials and Methods

### 2.1. Electrochemical Anodization of Nanostructured PSi

Nanostructured porous silicon was prepared by an electrochemical anodization process. Monocrystalline one-side-polished p-type silicon wafers with (100) orientation and resistivity in the 0.01–0.02 Ω.cm range were employed. A schematic design of the anodization process is shown in a previous study [15]. The etching solution is a 1:2 HF (48 wt %): ethanol (98 wt %) mixture. The anodization current density is 60 mA/cm^2^, and three different anodization periods were investigated as 18, 28, and 40 s.

### 2.2. Infiltration of AgNPs into Nanostructured PSi

The growth of silver nanoparticles was carried out by an electrochemical infiltration process. Nanostructured PSi samples with three different thicknesses were used as templates for the infiltration process. This process was performed using a Bio-Logic SP-150 potentiostat at a fixed current density of 1 µA/cm^2^ and infiltration time of 4 min. The aqueous solution composes of silver nitrate (0.1 mM), sodium citrate (0.25 mM), and nitric acid (0.01 M), with pH = 3.

### 2.3. Schottky Junctions’ Fabrication

**Figure 1:**
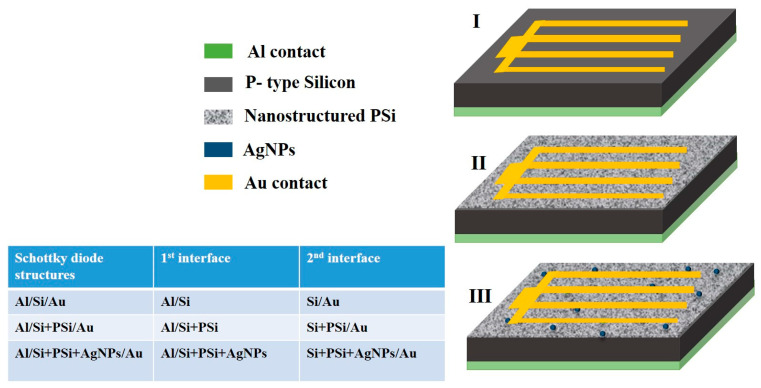
Schematic view of the structure of the fabricated diodes and a representation of the electrical interfaces between each layer: (I) Al/Si/Au diodes, (II) Al/Si + PSi/Au diodes, and (III) Al/Si + PSi + AgNPs/Au diodes.

Briefly, 100 nm thick Al back electrodes were deposited by electron beam evaporation. The base pressure was 2.5 $\times \;10^{-5}\;$mbar and the evaporation time was 2 min. Au top contacts were fabricated by DC magnetron sputtering using a metallic mask to define their geometry at the microscale. The evaporation process was carried out at a pressure of 5$\times \;10^{-2}$ mbar and for 4 min. Accordingly, Al/Si/Au is the base Schottky diode structure. This structure was modified by the addition of PSi and PSi + AgNPs, leading to Al/Si + PSi/Au and Al/Si + PSi + AgNPs/Au devices, respectively. The structure of the three Schottky junctions is schematically shown in Figure 1, in addition to a table portraying a description of the electrical interfaces for each junction.

### 2.4. Characterization Techniques

#### 2.4.1. Morphological Characterizations

Top-view and cross-sectional images of every sample were acquired using a Philips XL 30S-FEG field-emission scanning electron microscope (FESEM) (XL-40 FEG, Philips, Eindhoven, The Netherlands). The dimensions of the nanopores of PSi and AgNPs were determined using the free ImageJ software (Wayne Rasband at the National Institutes of Health, Bethesda, MD, USA).

#### 2.4.2. Optical Reflectance Characterizations

The optical reflectance of the fabricated PSi and hybrid PSi + AgNPs was carried out using a double-beam spectrophotometer (V-560, JASCO International, Tokyo, Japan) equipped with an integrated sphere to collect both diffuse and specular reflectance, with unpolarized light being almost normal to the sample. The angle of incidence was 10°.

#### 2.4.3. Electrical Characterizations

Alternating current (AC) and direct current (DC) measurements were carried out for Si-based metal–semiconductor diodes. The electrical measurements were carried out using a Bio-Logic (SP-150, Seyssinet, Pariset, France) potentiostat instrument. DC measurements were studied through current–voltage measurements (I–V) curves in the voltage range from −1.5V to 1.5 V at a scan rate of 20 mV/s. In addition, the AC measurements were carried out using electrochemical impedance spectroscopy (EIS) in a frequency range of 1 HZ to 1 MHZ, and a voltage amplitude of 0.5 V was applied. All measurements were carried out at room temperature in a Faraday cage, to shield the electrical measurements from external signals.

#### 2.4.4. Photoresponse Characterizations

The spectral responsivity in the 300 to 1200 nm wavelength range was determined at 0 V bias using a dual digital lock-in amplifier (Signal Recovery 7225-Wokingham, UK) at a chopper frequency of 300 Hz. Illumination was provided by an Acton Research Corporation Tungsten–Deuterium dual light source (model TDS-429, Wokingham, UK) and a (SpectraPro 150 monochromator, Wokingham, UK) equipped with two interchangeable diffraction gratings (1200 lines per mm) was used to select the wavelength.

## 3. Results

### 3.1. Morphology

**Figure 2:**
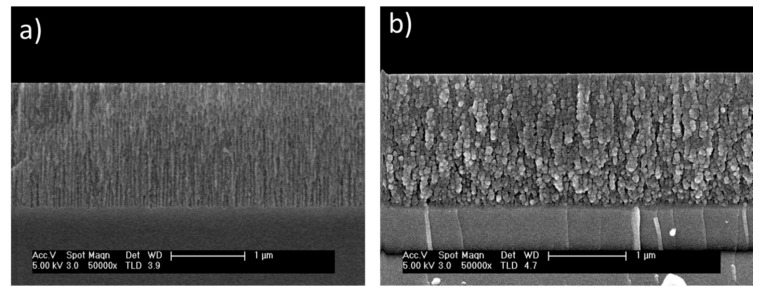
A cross-sectional view of a typical morphology of nanostructures Psi: (**a**) before infiltration of AgNPs and (**b**) after infiltration of AgNPs for 4 min.

The etching of silicon wafers leads to the formation of nanopores in the heavily doped regions [8]. The size of the nanopores depends on the anodization current density, while the thickness increases with increasing anodization time [16,17]. Figure 2a shows the FESEM cross-sectional view of nanostructured columnar porous silicon. The PSi layers are fabricated under an anodization current density of 60 mA/cm^2^ for 40 s. The thickness of the PSi layers is around 1400 nm, with typical pore diameters in the range of tens of nanometers. Upon increasing the etching time, the thickness of the PSi layer increases. Furthermore, Figure 2b shows a FESEM cross-sectional view of the typical PSi layers after infiltration of AgNPs into the nanopores. It can be observed that the size of the AgNPs is in the range of 10 nm to 15 nm. Figure 2b confirms the infiltration of the nanoparticles inside the nanopores. 

**Figure 3:**
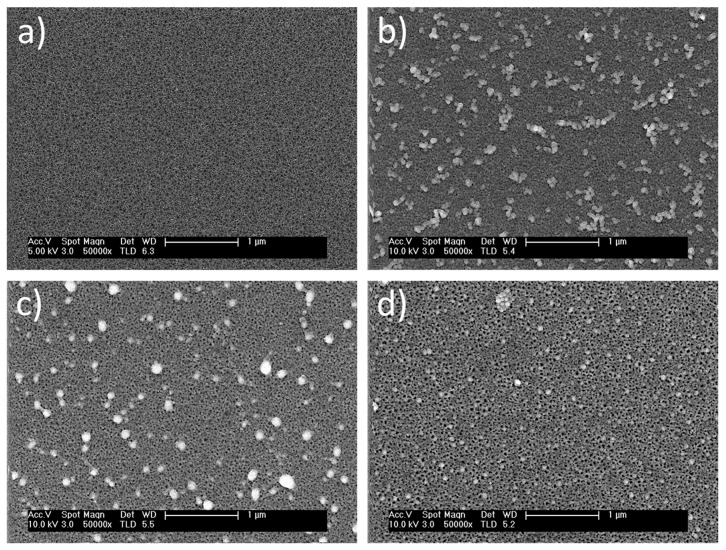
FESEM top-view images of a typical nanostructured PSi and hybrid PSi + AgNPs layers: (**a**) PSi top-view image (40 s etching time), (**b**) hybrid PSi + AgNPs top-view image (18 s etching time), (**c**) hybrid PSi + AgNPs top-view image (28 s etching time), and (**d**) hybrid PSi + AgNPs top-view image (40 s etching time). PSi etching current density of 60 mA/cm^2^ and AgNPs infiltration conditions are constant.

The density of infiltrated AgNPs inside the nanopores of porous materials usually depends on their diameter and thickness. In the current study, three sets of samples of PSi were etched at the same current density and for different etching times (18, 28, and 40 s). As a result, PSi layers of thicknesses 400, 1000, and 1400 nm, respectively, were obtained. Figure 3 shows the top-view images of nanostructured PSi and hybrid PSi + AgNPs with different thicknesses of PSi layers and the same density of AgNPs (infiltrated for the same period, 4 min). Figure 3a shows a typical top-view image of the PSi layers. In addition, Figure 3b–d show the top surface of the PSi layers after infiltration of AgNPs. The density of AgNPs is the same in the three samples. However, the thickness of the PSi layers increases from 400 nm to 1400 nm. Accordingly, the density of the nanoparticles that grew inside the nanopores increases with increasing the thickness of the PSi layer.

### 3.2. Overall Reflectance

**Figure 4:**
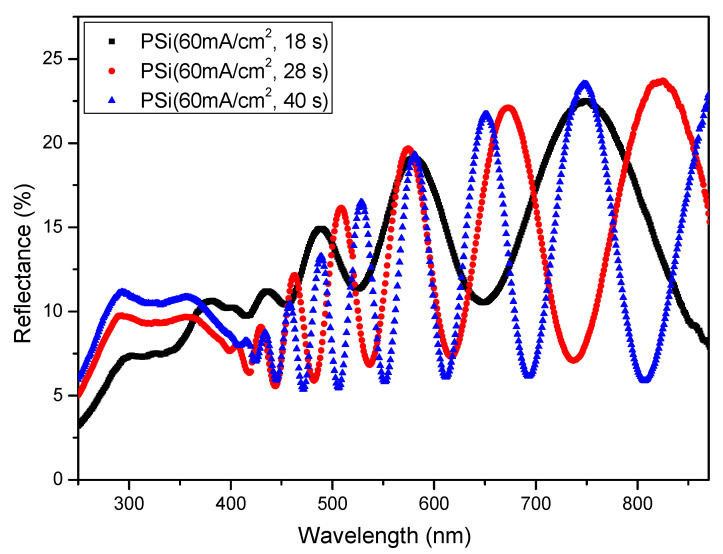
Overall reflectance of typical nanostructured PSi samples as a function of the thickness of PSi layer.

The use of porous silicon is known as a suitable way to reduce the optical reflectance of silicon [4]. This optical property is due to the morphology of the PSi layer, which is a composite of silicon nanocrystallites, amorphous silicon, and air [9,10]. Moreover, its optical property depends on the porosity and thickness of the porous layer [6]. Figure 4 illustrates the reduction in the optical reflectance due to the increase in the etching time (increased thickness) of the PSi layers.

**Figure 5:**
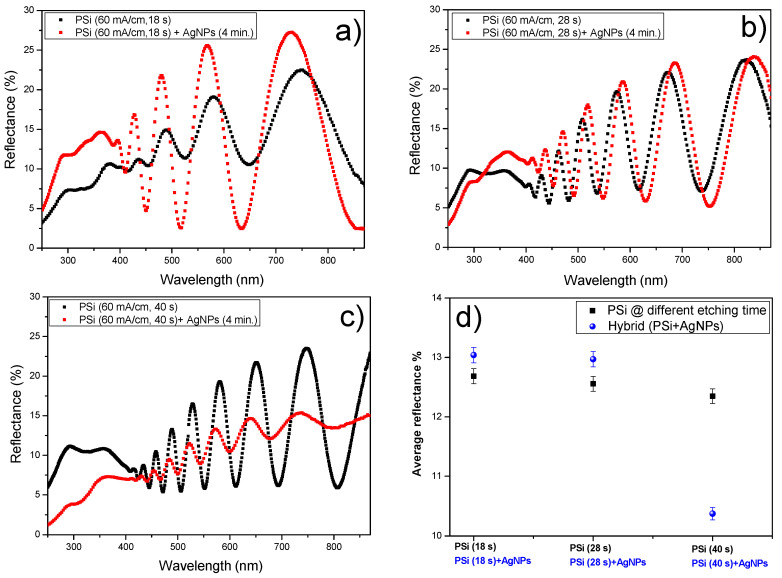
Overall reflectance property of PSi and hybrid PSi + AgNPs: (**a**) the thickness of PSi layer is 400 nm, (**b**) the thickness of PSi layer is 1000 nm, (**c**) the thickness of PSi layer is 1400 nm, and (**d**) comparison between the average reflectance of PSi and PSi + AgNPs at different thicknesses of PSi layers. AgNPs infiltrated at the same conditions.

Additionally, metallic nanoparticles might enhance the optical absorption of photonic and plasmonic devices [18,19]. Figure 5 shows a comparison between the optical reflectance of PSi and hybrid PSi + AgNPs layers as a function of the thickness of the PSi layer. The size, shape, and density of infiltrated AgNPs are the same. The optical properties of hybrid PSi + AgNPs nanostructures depend on the density of the infiltrated metal nanoparticles. Figure 5a shows the increase in the optical reflectance for PSi + AgNPs nanostructures. This effect is attributed to the large density of AgNPs on the surface of the PSi layer. However, the increase in the thickness of the PSi layer leads to growing more nanoparticles inside the nanopores. As a result, this leads to a reduction in optical reflectance, as illustrated in Figure 5b,c. Furthermore, Figure 5d shows the difference between the average reflectance of PSi and hybrid PSi + AgNPs for different PSi thicknesses. Thus, increasing the thickness of the PSi layer to 1400 nm leads to a 20% improvement in the optical absorption of hybrid nanostructures, while a notable increase in the average reflectance of hybrid PSi + AgNPs nanostructures is observed at lower thicknesses of the PSi layer. This behavior is related to the large density of AgNPs on the PSi surface.

### 3.3. Electrical Measurements

#### 3.3.1. DC Electrical Properties

Modifying the structure of materials is generally carried out to alter their optical and electrical properties. Given that PSi is a composite of silicon nanocrystallites, amorphous silicon, and air, increased porosity would result in a reduction in its electrical conduction. The incorporation of metallic nanoparticles into a porous structure would, at least partially, compensate for the increased electrical resistance. Accordingly, the electrical characteristics (AC and DC measurements) for Schottky junction diodes shown in the scheme in Figure 1 were studied.

**Figure 6:**
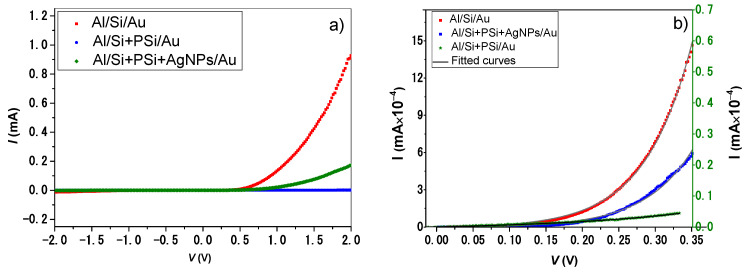
Electrical DC measurements in dark state: (**a**) current–voltage curves for the three Schottky diodes under forward and reverse biasing; (**b**) experimental current–voltage data under forward biasing and fitting to the modified diode model to extract the electrical parameters of Schottky diodes.

Figure 6 depicts the typical current–voltage curves for the fabricated metal–semiconductor diodes in the dark. The obtained results confirm the Schottky junction behavior for the three diodes. It is noted that the forward current increases with applying sufficient voltage, and the reverse current is ignoble. Moreover, the modified Al/Si + PSi/Au diode shows a degradation in electrical conduction, compared with the basic Al/Si/Au structure. This behavior is attributed to the formation of nanopores in the heavily doped regions of silicon during the etching process. Al/Si + PSi + AgNPs/Au diodes show a remarkable improvement in electrical conduction due to the incorporation of metallic nanoparticles. Therefore, the incorporation of metallic nanoparticles is a suitable method of combination, enhancing the optical and electrical properties of Schottky barrier diodes based on porous structures.

The electrical parameters of photonic devices can be used to predict the overall optoelectronic performance. These include parasitic resistances (series and shunt resistances), ideality factor, reverse saturation current, and barrier height. The electrical parameters of three Schottky configurations were accurately extracted by fitting the current–voltage curves to the non-ideal diode equation [20] as follows:

(1) $$I=I_{0}\left[e^{\frac{q\left(V-IR_{s}\right)}{nk_{B}T}}-1\right]+\frac{V-IR_{s}}{R_{sh}}$$

where *I* is the current in the dark state, *I_0_* is the reverse saturation current, n is the diode ideality factor, $R_{s}\;$is the series resistance, $R_{sh}$ is the shunt resistance, *k_B_* is the Boltzmann constant, and *T* is the absolute junction temperature.

Additionally, the barrier height can be calculated from the following equation [21]:

(2) $$\varphi_{B}=\frac{K_{B}T}{q}\ln\left(\frac{AA^{*}T^{2}}{I_{0}}\right)$$

where *A* (cm^2^) is the active area of the diodes, and A* is the effective Richardson constant, which takes the value 32 *A/*cm^2^K^2^ for p-type Si. 

Figure 6b confirms the accurate fitting of the experimental data to the diode equation. Table 1 presents the obtained electrical parameters. From the obtained results, it is concluded that the Al/Si + PSi/Au diode shows the lowest value of reverse saturation current (8.5098 ×$\;10^{-7}$ mA). This behavior might be related to the overall reduction in the electrical conduction of the Si + PSi layer. Additionally, the same diode yields the largest ideality factor (7.73) and barrier height (0.82) as a consequence of an increase in the PSi surface-to-volume area. To this end, the number of defects increases, and rapid oxidation of the PSi layer occurs [7]. Regarding the modified hybrid Al/Si + PSi + AgNPs/Au diodes, the ideality factor improved to 2.59. This enhancement is attributed to two main factors: (1) the passivation of the PSi layer by the chemical interaction with metal ions during the electrodeposition of the AgNPs and (2) the remarkable electrical enhancement by the AgNPs. Therefore, the number of defects due to the nanoholes is reduced. In the same manner, the series resistance improved after incorporation with AgNPs, in comparison with flat Si. This effect is attributed to the improvement in the adhesion with the front Au contact, and the electrical reduction by the PSi layer leads to an increase in the series resistance for Al/Si + PSi/Au diodes. The small current values under reverse bias (of the order of 10^−9^ A) indicate that *R_sh_* is very high for the three MIS Schottky barrier diodes. Accordingly, the last part of the diode equation can be safely neglected from the fitting process. The current transport in metal–semiconductor contacts is mainly due to the majority carriers [22]. According to the elaborate criterion derived by Sze and Rhodrick [22,23], four main factors confirm considering thermionic emission as the transport mechanism for the Schottky junctions: (1) The barrier height of the diodes is much larger than the thermal energy ($K_{B}T$ ~ 0.025 eV), as depicted in Table 1; (2) the transport mechanism can be described by the thermionic emission theory due to the high mobility of Si; (3) the existence of a net current flow does not affect the thermal equilibrium; (4) the generation of carriers is from the semiconductor over the potential barrier into the metal. 

#### 3.3.2. AC Electrical Properties

**Figure 7:**
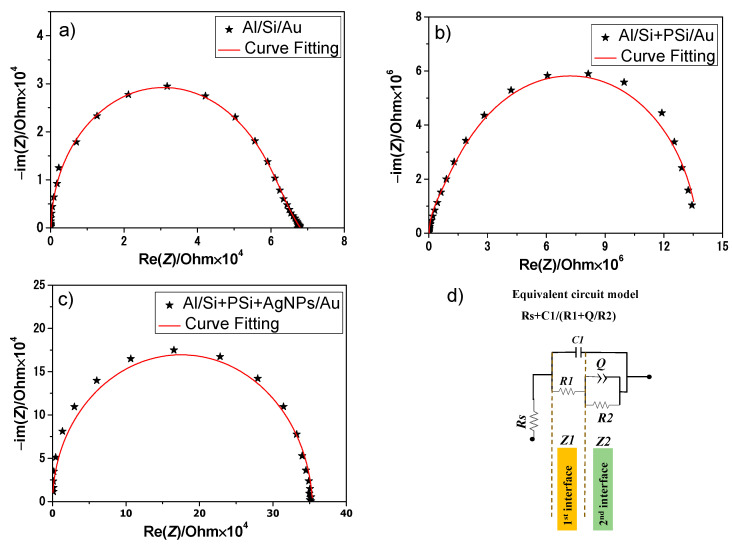
Nyquist plots for the performed Schottky diodes: (**a**) corresponds to Al/Si/Au diodes; (**b**) corresponds to Al/Si + PSi + AgNPs/Au diodes; (**c**) corresponds to Al/Si + PSi/Au diodes; (**d**) the favorable equivalent circuit model.

Impedance spectroscopy is an effective technique to study the electrical performance of the interfaces of electronic devices in a wide range of frequencies [14,24,25,26]. Figure 7 shows the Nyquist plots for the Al/Si/Au, Al/Si + PSi/Au and Al/Si + PSi + AgNPs/Au Schottky junctions. The *RC* electrical elements were obtained by fitting the Nyquist plots to the equivalent circuit model. Figure 7d portrays the used equivalent circuit model for the three structures of Schottky diodes. The equivalent model composes of series resistance and two parallel RC circuits. Accordingly, the semicircle at low frequency in Figure 7a–c is attributed to Al/Si, Al/Si + PSi, and Al/Si + PSi + AgNPs interfaces, respectively, while the second semicircle at high frequencies corresponds to the interfaces Si/Au, Si + PSi/Au, and Si + PSi + AgNPs. Si + PSi composes of heterostructures of two p-type semiconductor materials with different energy bandgaps [27]. Therefore, it was assumed that Si + PSi was considered as one layer. Moreover, the infiltration of AgNPs was inside and on the surface of the PSi layer. Therefore, Si + PSi + AgNPs was considered as one layer. 

Table 2 summarizes a comparison between the electrical parameters for the different Schottky junctions. The presence of the PSi layer leads to a remarkable reduction in the series resistance and electrical conductions of the first and second interfaces. For instance, the series resistance increases from 276.8 Ω to 19,333 Ω and R1 increased from 5.9 $\times \;10^{4}$ Ω to 3.36 $\times \;10^{6}$ Ω. This electrical reduction may be attributed to the morphology of the PSi layer, as discussed in Section 3.1 and Section 3.3.1 Furthermore, the increase in the surface-to-volume ratio of the PSi layer leads to an increase in surface states and electrical losses. On the other hand, a notable electrical enhancement by metallic AgNPs embedded into the PSi layer in the Al/Si + PSi + AgNPs/Au diodes, as shown in Table 2.

The inhomogeneous structure and roughness of the surface of the second interface lead to anomalous capacitive behavior. Therefore, a constant phase element (CPE) parallel to a resistor is the suitable equivalent circuit model for this interface. Thus, its characteristic capacitor can be depicted by the following equation [25]:

(3) $$C=Q^{\frac{1}{a}}\cdot R^{\frac{1-a}{a}}$$

where *Q* is the CPE and the factor a is an index that indicates the degree of imperfection of this element. This index can vary between 0 and 1, with 0 describing a perfect resistor, and 1 a perfect capacitor [28]. The large decrease in the values of *C1* and *C2* for the two interfaces of Al/Si + PSi/Au diodes might be attributed to the particular structure of the PSi layer. The increase in the surface-to-volume ratio of the PSi layer leads to an increase in the leakage current. However, incorporation with AgNPs in the Al/Si + PSi + AgNPs/Au Schottky diodes result in higher values of *C1* and *C2* for the two interfaces.

The minority carrier lifetime (*τ*) for every interface can be obtained from the following well-known relationship [29]:

(4) τ=RC

As a result of the progress in electrical conduction by the hybrid nanostructures of PSi + AgNPs, the minority carriers’ lifetime *τ1* (10.6 µs) and *τ2* (287.4 µs) decrease for Al/Si + PSi + AgNPs/Au Schottky diodes. The results are compiled in Table 2. Al/Si + PSi/Au diodes show the highest values of *τ1* and *τ2* (201 and 895 µs, respectively). This behavior is related to conduction losses by the porous structure of PSi layers and the formation of defects on their surface [7]. Additionally, the minority carrier diffusion coefficient can be calculated using the following equation [30]:

(5) $$D=\frac{L^{2}}{\;\tau }$$

where *L* is the average length for the carriers between generation and recombination [6]. As a result of the lowest values of *τ1* and *τ2* (10.6 and 287 µs, respectively) for Al/Si + PSi + AgNPs/Au diodes, diffusion length between generation and recombination for the corresponding two interfaces is large. Therefore, hybrid nanostructures of PSi + AgNPs will enhance the generated photocurrent for illuminated photodetectors.

### 3.4. Photoresponse Characteristic

**Figure 8:**
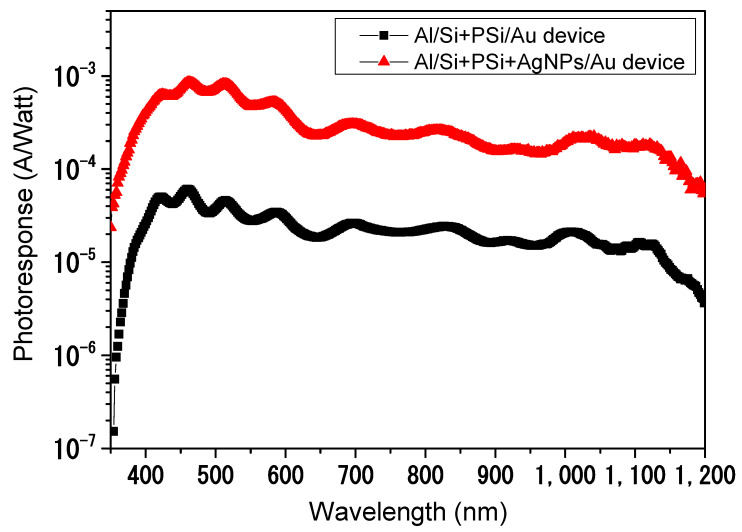
Comparison between the spectral photoresponse of Al/Si+PSi/Au and Al/Si + PSi + AgNPs/Au Schottky photodiodes at zero biasing.

Porous silicon can be described as a network of nanometer-sized silicon nanocrystallites surrounded by void space. Therefore, it can be used to improve the optical anti-reflective character of flat silicon. However, PSi oxidizes very fast in contact with air, which leads to rapid degradation in its performance, as discussed regarding the optical and electrical characteristics in Section 3.2 and Section 3.3. Incorporation by metallic nanoparticles can significantly improve the performance and stability of optoelectronic devices [3,7,8,9]. Figure 8 shows the spectral photoresponse of Al/Si + PSi/Au and Al/Si + PSi + AgNPs/Au photodiodes in the wavelength range from 300 nm to 1200 nm. The spectral photoresponse was obtained by dividing the generated photocurrent by the spectral emissive power of the incident light source. From the experimental results, the generated photocurrent from Al/Si + PSi + AgNPs/Au photodiodes is approximately 10 times higher than the one generated from Al/Si + PSi/Au photodiodes in the broad range of the electromagnetic spectrum. Accordingly, infiltration of AgNPs inside nanostructured PSi leads to a remarkable improvement in the photoresponse of the photodiodes in this study. Three main factors are concluded due to this optoelectronic enhancement by hybrid PSi + AgNPs nanostructures: (1) passivation of nanostructured PSi layers occurs during the infiltration of AgNPs through chemical interaction with a noble metal; (2) infiltration of AgNPs partially prevents nanostructured PSi from oxidation, which leads to a drastic reduction in surface recombination due to oxygen vacancies; (3) growing AgNPs into the PSi layer leads to an overall improvement in the electrical conduction of photodiodes.

## 4. Conclusions

We analyzed the effect of etching time on the optical properties of electrochemically etched PSi layers and hybrid Psi + AgNPs nanostructures. From the experimental results, it is noted that the increase in the etching time of the PSi layer leads to an enhancement in optical absorption. In particular, increasing the thickness of PSi layers to 1400 nm leads to a 20% improvement of the optical absorption of hybrid nanostructures. A notable increase in the average reflectance of hybrid PSi + AgNPs nanostructures at lower thicknesses of the PSi layer is also observed.

As a consequence of the morphology of PSi layers, a reduction in electrical conduction should be observed with increasing porosity. The incorporation of metallic nanoparticles into the porous structure compensates for the reduction in electrical conduction. The electrical characteristics (AC and DC measurements) for three Schottky junction diodes (Al/Si/Au, Al/Si + PSi/Au and Al/Si + PSi + AgNPs/Au) were studied, and their electrical parameters were determined. Regarding electrical conduction, the modified Al/Si + PSi/Au diodes show degradation in electrical conduction, compared with the basic Al/Si/Au structure. However, tAl/Si + PSi + AgNPs/Au diodes show a remarkable improvement in electrical conduction due to the incorporation of metallic nanoparticles. 

Additionally, the photoresponse of modified Schottky junctions was studied in wavelengths in the range of 300 nm to 1200 nm. From the experimental results, the generated photocurrent from Al/Si + PSi + AgNPs/Au photodiodes is found to be approximately 10 times higher than the one generated by Al/Si + PSi/Au photodiodes in a broad wavelength range. Accordingly, the infiltration of AgNPs inside nanostructured PSi leads to a remarkable improvement in the photoresponse of photodiodes. Three main factors are considered to be responsible for the observed optoelectronic enhancement by hybrid Psi + AgNPs nanostructures: (1) passivation of nanostructured PSi layers occurs during infiltration of AgNPs through chemical interaction with a noble metal; (2) infiltration of AgNPs partially prevents nanostructured PSi from oxidation, which leads to a drastic reduction in surface recombination due to oxygen vacancies; (3) growing AgNPs into the PSi layer leads to an overall improvement in the electrical conduction of the photodiodes.

## Figures and Tables

**Table 1:** DC electrical parameters obtained from the fitting of the experimental I–V curves.

Device Structure	*I*_0_ (mA)	*n*	*Rs* (kΩ)	*φ_B_* (V)
Al/Si/Au	$6.15982\;\times \;10^{-6}$	2.55	0.105	0.77
Al/Si + PSi/Au	$8.5098\;\times \;10^{-7}$	7.73	1.478	0.82
Al/Si + Psi + AgNPs/Au	$2.77652\;\times \;10^{-6}$	2.59	0.043	0.79

**Table 2:** Comparison between the electrical parameters between the interfaces of the three Schottky diodes studied in this study.

Calculated Parameter		Schottky Diode Structure		
		Al/Si/Au	Al/Si + PSi/Au	Al/Si + PSi + AgNPs/Au
*R_s_* (Ω)		276.8	19333	400
*C1* (nF)	1st Interface	1.16	$59.96\;\times \;10^{-3}$	$31.36\;\times \;10^{-3}$
*R1* (Ω)		$5.9\;\times \;10^{4}$	$3.36\;\times \;10^{6}$	$3.4\;\times \;10^{5}$
*τ1* (µs)		68.4	201.46	10.6
*Q* (f.s^a−1^)	2nd Interface	$0.44\;\times \;10^{-6}$	$0.187\;\times \;10^{-9}$	$0.11\;\times \;10^{-6}$
*a*		0.66	0.89	0.58
*C2* (nF)		40.52	$86.4\;\times \;10^{-3}$	$95.8\;\times \;10^{-2}$
*R2* (Ω)		$2.09\;\times \;10^{4}$	$10.36\;\times \;10^{6}$	$1.3\;\times \;10^{4}$
*τ2* (µs)		846.8	895.1	287.4
